# Tremorgenic Mycotoxins: Structure Diversity and Biological Activity

**DOI:** 10.3390/toxins11050302

**Published:** 2019-05-27

**Authors:** Priyanka Reddy, Kathryn Guthridge, Simone Vassiliadis, Joanne Hemsworth, Inoka Hettiarachchige, German Spangenberg, Simone Rochfort

**Affiliations:** 1 Agriculture Victoria, AgriBio, Centre for AgriBioscience, Bundoora, Victoria 3083, Australia; Priyanka.reddy@ecodev.vic.gov.au (P.R.); Kathryn.guthridge@ecodev.vic.gov.au (K.G.); simone.vassiliadis@ecodev.vic.gov.au (S.V.); Joanne.hemsworth@ecodev.vic.gov.au (J.H.); Inoka.Hettiarachchige@ecodev.vic.gov.au (I.H.); german.spangenberg@ecodev.vic.gov.au (G.S.); 2 School of Applied Systems Biology, La Trobe University, Bundoora, Victoria 3083, Australia

**Keywords:** mycotoxins, endophyte, fungi, neurotoxin, lolitrems

## Abstract

Indole-diterpenes are an important class of chemical compounds which can be unique to different fungal species. The highly complex lolitrem compounds are confined to *Epichloë* species, whilst penitrem production is confined to *Penicillium* spp. and *Aspergillus* spp. These fungal species are often present in association with pasture grasses, and the indole-diterpenes produced may cause toxicity in grazing animals. In this review, we highlight the unique structural variations of indole-diterpenes that are characterised into subgroups, including paspaline, paxilline, shearinines, paspalitrems, terpendoles, penitrems, lolitrems, janthitrems, and sulpinines. A detailed description of the unique biological activities has been documented where even structurally related compounds have displayed unique biological activities. Indole-diterpene production has been reported in two classes of ascomycete fungi, namely Eurotiomycetes (e.g., *Aspergillus* and *Penicillium*) and Sordariomycetes (e.g., *Claviceps* and *Epichloë*). These compounds all have a common structural core comprised of a cyclic diterpene skeleton derived from geranylgeranyl diphosphate (GGPP) and an indole moiety derived from tryptophan. Structure diversity is generated from the enzymatic conversion of different sites on the basic indole-diterpene structure. This review highlights the wide-ranging biological versatility presented by the indole-diterpene group of compounds and their role in an agricultural and pharmaceutical setting.

## 1. Introduction

**Figure 1:**
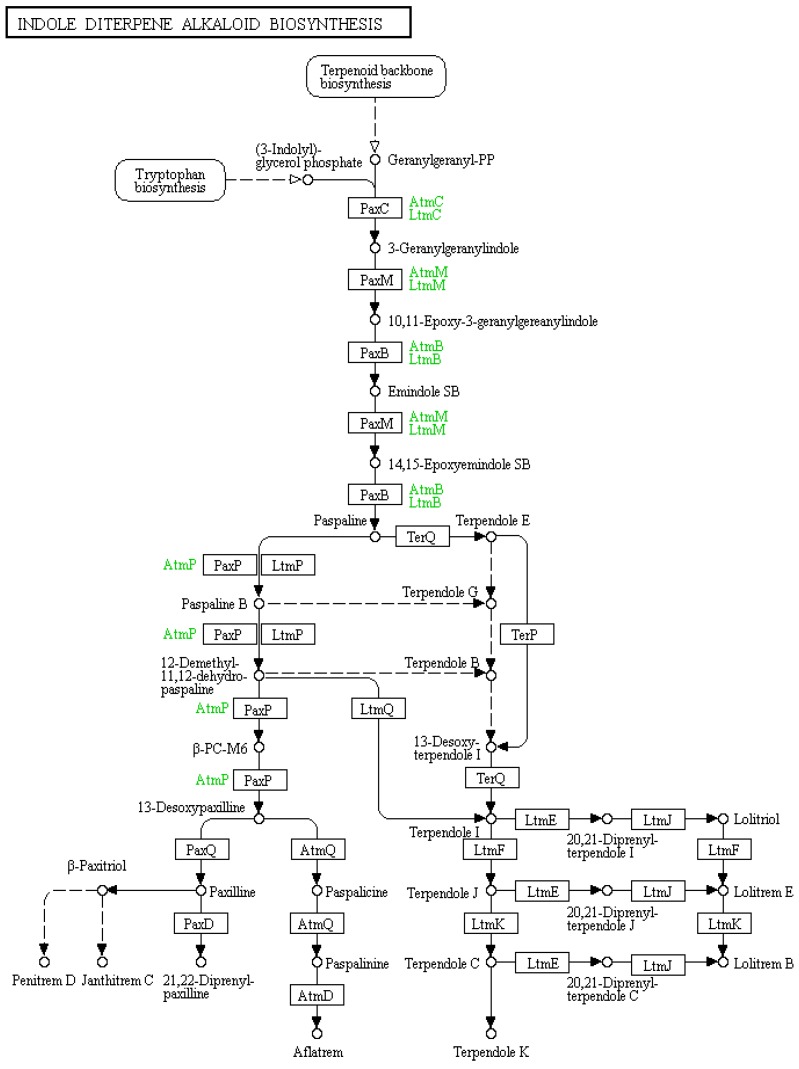
Indole-diterpene biosynthetic map sourced from KEGG pathways showing the enzymes responsible for producing indole-diterpene compounds at each step. Reproduced from [29,30,31].

Perennial ryegrass (*Lolium perenne* L.) is used for forage in temperate regions throughout the world including Northern Europe, Pacific North West of USA, Japan, South-eastern Australia, and New Zealand [1,2,3,4,5]. It is the most commonly utilized pasture grass on dairy farms in Australia and has a high economic importance [6]. The asexual form of the endophytic fungus *E. festucae* var. *lolii* (previously known as *Neotyphodium lolii* and *Acremonium lolii*) is known to establish a symbiotic relationship with perennial ryegrass [7]. These interactions are beneficial to the pastoral agriculture industry as compounds produced by the endophyte confer resistance to biotic and abiotic stresses. For example, a select number of indole-diterpene class compounds, such the lolitrems A, B, and E that are present in endophyte-infected ryegrasses, are toxic to the larvae of the Argentine stem weevil (*Listronotus bonariensis*) [8,9]. The grass–endophyte association also produces secondary metabolites that are detrimental to grazing animals. The major toxins of concern are the alkaloids ergovaline and lolitrem B, which are present in old naturalized perennial ryegrass pastures containing the Standard Endophyte (SE) strain. Although both toxins are produced by endophyte-infected perennial ryegrass, ergovaline is normally most abundant in endophyte-infested tall fescue grass and causes the vasoconstrictive conditions fescue foot or summer slump disease [10,11]. The indole-diterpenes are predominant in endophyte-infected perennial ryegrass and lolitrem B is the end-point of the complex indole-diterpene biochemical pathway (Figure 1) [12]. Many of the indole-diterpene class of compounds, particularly the lolitrems, are reported as anti-mammalian alkaloids that significantly affect animal health. In particular, lolitrem B has been identified as a causative agent for perennial ryegrass staggers disease, a nervous disorder, notably of sheep and cattle that causes tremors [12]. However, despite the prevalence of perennial ryegrass in various geographic locations the toxicity reports are generally limited. Neurological signs associated with ryegrass staggers disease has been reported in animals, particularly sheep, grazing on perennial ryegrass in Australia, New Zealand, as well as Pacific northwest of USA and Europe [13,14]. This imposes a negative impact on industry, particularly dairy, meat, and wool production involving grazing animals [15]. Thus, forage improvement programs have been involved in selecting novel endophytes that do not produce the known toxins; however, complex mixtures of the intermediate compounds are still present in marketed forage grasses.

Symptoms of ryegrass staggers include initial head tremors, muscle fasciculation of the neck and legs, and hypersensitivity to external stimuli as a result of the neurotoxic effect. To date, no physical effects or gross lesions have been reported, and animals completely recover when the toxin is eliminated from their systems. Although, affected animals suffer poor weight gain and they may be difficult to handle due to their hypersensitivity [16,17,18]. Death is rarely a consequence unless an accident, such as drowning, occurs during an episode in which the animal loses voluntary control [18]. One of the methods to control the disease centers on offering alternative feed sources or removal of animals from drought-stressed and overgrazed pastures during summer and early autumn when the toxin levels are elevated [19] and neurological signs appear [18]. 

Lolitrem B is the most abundant of the indole-diterpene series of compounds produced by perennial ryegrass endophytes belonging to *Epichloë festucae* var. *lolii* (termed *Lp*TG-1). Lolitrem B has many analogues and precursors that are also known to elicit tremors in animals (Table 1) [20,21,22]. Although the structure–activity relationship is unclear, the literature suggests that lolitrem analogues, and biosynthetic intermediates such as paxilline and terpendole C, cause tremors in grazing animals [22]. Penitrems have also shown to exhibit clinical signs that are similar to ryegrass staggers disease, though intoxication is commonly documented as a result of exposure to moldy foods [23,24]. Additionally, it would be unwise to assign a single compound as the causative agent when a complex mixture of related compounds exists in grass–endophyte associations. Typically, the presence and absence of the major compounds are used in the screening of endophyte-infected grasses and the effect of individual intermediate compounds and their synergistic effects are largely ignored [25]. Furthermore, some of the indole-diterpenes produced could be innocuous to grazing animals and beneficial in deterring insects at the same time [15]. This is an important factor to consider when novel grass–endophyte associations are in development.

There is a growing need to understand the toxicity of all compounds within the indole-diterpene group, including intermediates and analogues, as shown in the biosynthetic map in Figure 1. However, obtaining these compounds to test on animal models is challenging due to the difficulty involved in compound isolation and purification such that large kilogram (kg) quantities of starting material is required to obtain milligrams of some of the intermediates [26,27]. For example, Munday-Finch et al. enriched fractions obtained from extraction of 360 kg of seed and were then able to identify lolicine A, lolicine B, lolitriol, and lolitrem N, as well as the naturally occurring 31-epilolitrem N and 31-epilolitrem F [27,28]. No biological activities were determined for these compounds other than 31-epi-lolitrem F [27]. Thus, it would be more effective to understand structure–activity associations as tools for predicting the toxicity of novel or previously uncharacterized compounds. 

The purpose of this review is to provide an exhaustive compilation of biological activities reported for indole-diterpene compounds produced by *Epichloë* endophytes. Where known, biological activities of some indole-diterpenes produced by other fungal genera such as *Aspergillus* and *Penicillium* are also presented. 

## 2. Reported Animal Toxicity for Indole-Diterpenes

In 1986, Gallagher and Hawkes established mouse model assays to assess the tremorgenicity of lolitrems using a visual rating scale and a positive control (lolitrem B or paxilline) [16]. The mouse model assay showed good correlation to large animal models as seeds, deemed toxic through mouse studies, were also neurotoxic to sheep that were orally fed with pellets containing the toxin [32]. Mouse model assays established by Gallagher and Hawkes were used to assess tremor intensity of most of the indole-diterpenes described in Table 1 [16].

Another technique to test toxicity in larger animal models is electromyography (EMG), a method for measuring and evaluating the electrical activity of muscles. McLeay et al. carried out toxicity studies on sheep in which EMG activity of skeletal muscles and the smooth muscles of the reticulum and rumen were measured, in response to single doses of penitrems (mixture of 88.3% penitrem A, 6.4% penitrem B, 5.3% penitrem E), paxilline, lolitrem B, and 31-epilolitrem B [33] (Table 1). It was found that the reticulum and rumen muscles showed inhibition of normal electrical activity, which coincided with the induction of tremoring associated with skeletal muscle activity in penitrems, paxilline, and lolitrem B [33]. These findings indicate that disruption of digestion may occur in animals grazing endophyte-infected pasture, especially in the case of lolitrem B, in which perturbations in muscle electrical activity lasted 12 h [33].

Studies to further understand the mode of action of indole-diterpenes were conducted based on evidence relating to potassium channel inhibition as a potential mechanism of tremorgens and observed symptoms of hyperexcitation of the central nervous system [34,35,36,37,38]. In particular, Big Potassium (BK) channel receptor inhibition was tested in response to a series of compounds, as shown in Table 1, since BK channels have major roles in smooth muscle function and neuronal excitability [39]. 

It has been reported that mice deficient in BK ion channels are unaffected by these neurotoxins at concentrations that are lethal to wild-type mice [37]. This suggests that motor function deficits induced by lolitrems are mediated by BK channels [37]. These BK channels are independently activated by depolarizing membrane voltages and elevated intracellular calcium and magnesium [39]. The BK channel is suggested as the major molecular target for these compounds as they are reported to cause inhibition of the BK channel currents. The indole-diterpenes show differences in their interaction with BK channels in vitro and these differences are also apparent when comparing in vivo response of these compound such as duration of tremor and effects on motor function. Paxilline shows BK current inhibition to be calcium-dependent and there is reduced inhibition with increased calcium concentration [40] which is reported to rightward shift the conductance–voltage (G–V) [40,41]. It is also later reported that the G–V shift induced by paxilline is dependent on calcium concentration and an open state preference for BK (*hSlo*) channels [39]. In contrast, inhibition by lolitrem B does not affect G–V relationship [41], Although, both compounds are reported to possess a calcium concentration-dependence to their inhibitory effects and show higher apparent affinity for the open state of the BK channel in comparison to the closed state [41]. 

The concentration at which half-maximal inhibition (IC_50_) is achieved is reported in Table 1. However, lolitrem E and paspalicine although showing potent BK channel activity, elicit a nontremorgenic effect on animals. This could be related to structural changes occurring in vivo, rendering it less active [41]. Knaus et al., suggested that although some pharmacological properties could be explained by BK channel inhibition, tremorgenicity may not be directly related to channel block [36]. 

**Figure 2:**
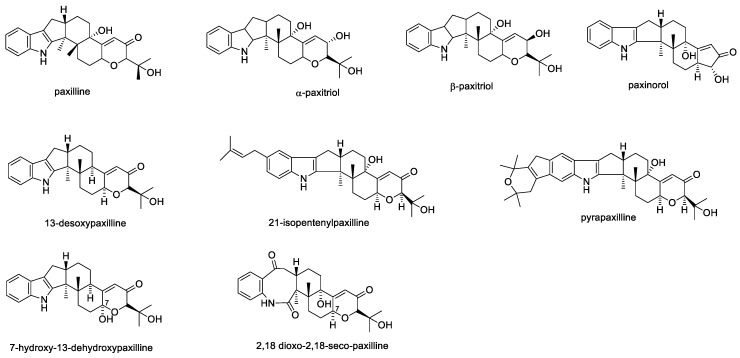
Structures of paxilline and selected derivatives.

Postulations regarding structure–activity relationship have been reported for motor and coordination deficits previously shown in mouse models and BK channel activity [37,39,41,42,43]. For example, the key attributes that have been postulated for the tremorgen lolitrem B as a potent neurotoxin include the presence of the acetal-linked isoprene unit on the right-hand side of the molecule, presence of the A/B rings and the position of the hydrogens in the junction of the rings (Figure 2). The presence of the A/B rings in particular are thought to be responsible for the slow-onset and long-acting tremorgenic activity of the lolitrem toxins [28,41]. Loss of the isoprene unit and opening of the ring attached to this isoprene unit removes the tremorgenic potency, as seen in lolitrem M and when lolitrem B loses its tremorgenicity when it degrades to lolitriol [28]. Table 1 describes in detail the results obtained from the assays described above for compounds belonging to indole-diterpene biosynthetic pathways.

### 2.1. Classes of Indole-Diterpenes and Their Reported Activities

#### 2.1.1. Paxilline

Paxilline is produced by many types of fungi and was originally identified in *Penicillium paxilli* [61]. In 1975, the structure of paxilline was elucidated and the biological activity tested in mice [62]. It was found to induce tremors that sustained for several hours, yet it was evidently less toxic than other tremorgens exhibiting a LD_50_ of 150 mg/kg body weight [62]. In comparison to lolitrem B, it is reported to produce shorter and less intense tremors in mice [43] and other vertebrate animals [63]. It is also a potent and selective BK channel inhibitor [36,40,64,65].

There are many other paxilline derivatives, such as α-paxitriol and β-paxitriol (Figure 2), which are proposed precursors of terpendoles and lolitrems as well as janthitrems and penitrems, respectively [66]. Structurally related compounds of paxilline are also reported to possess unique biological activities. For example, pyrapaxilline and 21-isopentenylpaxilline have been reported to inhibit the production of the neurotransmitter nitrogen monoxide (NO), though with less potency than paxilline [67]. The suppression of NO production is important for treating inflammatory diseases such as rheumatoid arthritis and atherosclerosis, a disease in which plaque builds up inside arteries [67]. Paxilline and paxilline derivatives were shown to be readily produced by various fungi in culture. For example, paxilline acetate, 13-desoxypaxilline (13-dehydroxypaxilline), from *Emericella striata* [68], 7-hydroxy-13-dehydroxypaxilline, 13-desoxypaxilline, 2,18-dioxo-2,18-seco-paxilline from *Eupenicillium shearii* [69] and paxinorol from *Penicillium paxilli* [70]. The structure of paxilline and several of its derivatives are indicated in Figure 2 and associated biological activities for mammalian toxicity (where known) are indicated in Table 1. 

#### 2.1.2. Lolitrems

**Figure 3:**
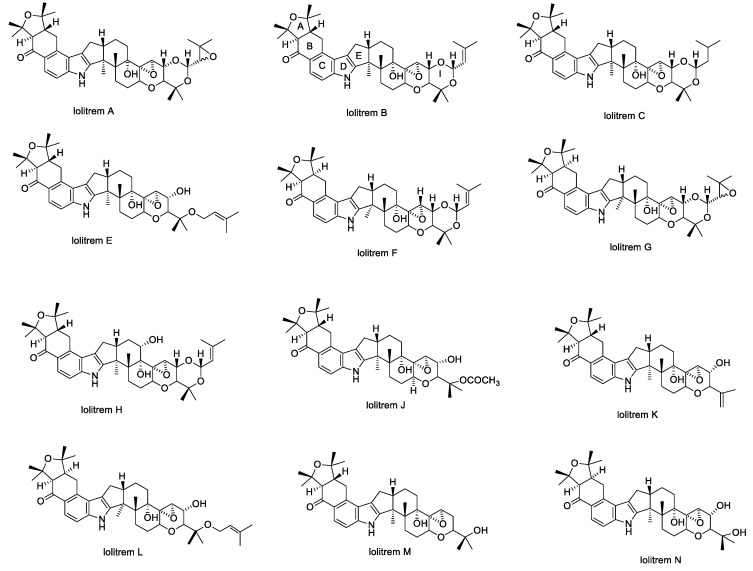
Structures of lolitrems A–N.

**Figure 4:**
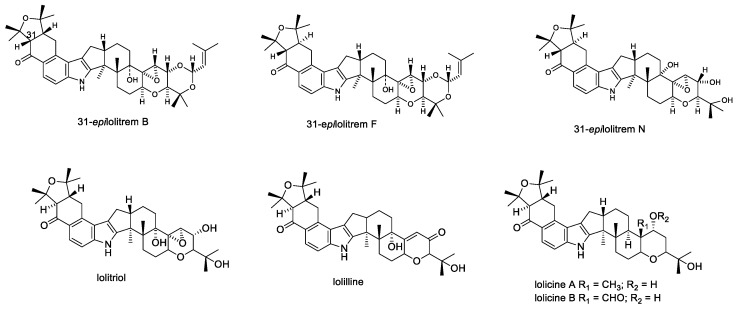
Structures of lolitrem derivatives.

In 1981, Gallagher et al. reported that lolitrems were the causative agents of ryegrass staggers in animals grazing perennial ryegrass pastures [71]. This study, and later reports, show that seeds containing lolitrems had the same toxic effect in symptomatology and the same reversible nature of neurotoxicity as observed for ryegrass staggers [32,71]. Later, in 1984, Gallagher reported the properties of lolitrem A–D and described full characterization studies of the major compound, lolitrem B [72]. Lolitrems A–N (Figure 3) and the derivatives epilolitrems, lolicines, and lolitriols (Figure 4) have been isolated and their structures elucidated [20,27,28,43,44,46,47]. The epi-lolitrems, are lolitrem B and lolitriol analogues which differ only in their stereochemistry at C-31 and C-35 position [27,46].

The prolonged duration of tremors elicited by lolitrem B and its complete reversible response is considered to be of pharmacological importance [16]. The mode of action of the indole diterpenes, mainly as BK channel inhibitors, have allowed them to be used as lead compounds for pharmaceutical drug discovery. 

Indole diterpenes, including nontremorgenic alkaloids have been reported to significantly inhibit the production of cytokines particularly those with a proinflammatory response. Cytokine proteins are produced by cells in response to an infection or trigger. The over-response of the immune system in humans to foreign substances, i.e., anaphylactic reaction, can cause fatalities. In the case of lolitrem B and 31-epi-lolitrem B, the production of cytokines IL-6 and TNFa was significantly inhibited in murine macrophage cells. It is also found that these compounds showed no toxicity against the host cells by means of cell proliferation assay at concentrations 100 and 250 times higher. Thus, it is suggested that these compounds would make good candidates for drug design [34]. 

#### 2.1.3. Penitrems

**Figure 5:**
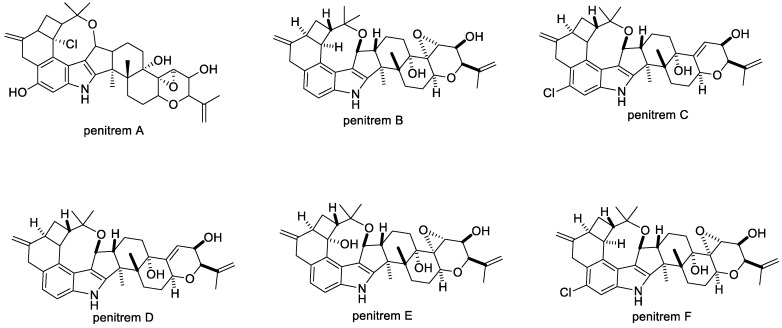
Structures of penitrems A–F.

Penitrems are found in several species of fungi and have been described particularly well in the case of *Penicillium crustosum* [73,74]. Penitrem A is the most tremorgenic and abundant of the penitrems (Figure 5) [54,75]. It causes severe sustained tremors in mice when given intraperitoneally at a dose of 1 mg/kg [52]; and in sheep when given intravenously at dose of only 20 µg/kg (Table 1). Furthermore, sheep dosed intravenously with 5.5 mg/kg of a penitrem mixture showed strong inhibition on the reticulum and rumen despite no significant skeletal EMG activity (Table 1). Penitrem A is also known to cause neuronal death, in particular of Purkinje neurons, located in the cerebellum, that are critical for coordination and movement [75]. 

Early work investigating the mode of action of the mycotoxins penitrem A and verruculogen shows that these toxins interfere with amino acid neurotransmitter release mechanisms in the central nervous system [76,77,78]. Penitrem A as well as another mycotoxin verruculogen are known to interfere with amino acid neurotransmitters release mechanisms in the central nervous system [76,77,78,79]. Norris et al. conducted a study in which toxins were administered in vivo and synaptosomes were subsequently prepared from cerebrocortical and spinal cord medullary regions of rat, and corpus striatum of sheep [77]. In this study, penitrem A increased and spontaneous release of glutamate [77], GABA, and aspartate from the cerebral cortex and midbrain region was observed [77]. However, no change was observed in the synthesis or release of dopamine and other amino acids and neurotransmitters in the central nervous system [77,78]. The tremorgen verruculogen increased the spontaneous release of glutamate and aspartate and decreased and inhibited uptake of GABA in mice in the central nervous system [77,78,80,81,82]. 

BK channels are important for cancer development and progression. Thus, BK channel blockers such as the indole diterpenes would be regarded as potential candidates for cancer therapeutic drugs [83,84,85]. Penitrems A, B, D, E, and F (Figure 5) as well as 6-bromopenitrem B and 6-bromopenitrem E are reported to show in vitro inhibition of proliferation, migration, and invasion properties against human breast cancer cells [86]. This effect was also observed for less complex biosynthetic intermediates emindole SB and paspaline [86]. These compounds were identified as potential candidates for future studies as they do not inhibit BK channels, thus eliminating any associated toxicity in relation to tremorgenicity [86]. A blockade of the α-subunit of the BK channels would also interfere with downstream biological functions such as neurotransmitter release and activation in the central and peripheral nervous system. A more targeted approach on β-catenin in human breast cancer cells was reported to have success with specific structural analogues of penitrems [87]. Additionally, combination therapy with other agents has been shown to reduce the associated toxicity [88]. Penitrem B also showed in vitro growth inhibition of the human tumor cell line screen representing leukemia [86]. This test was carried out using 60 cell lines representing various cancers (e.g., leukemia, melanoma, and cancers of the lung, colon, brain, ovary, breast, prostate, and kidney) [86]. Selective activity was exhibited by penitrem B against all cancer cells representing leukemia [86].

#### 2.1.4. Paspaline

**Figure 6:**
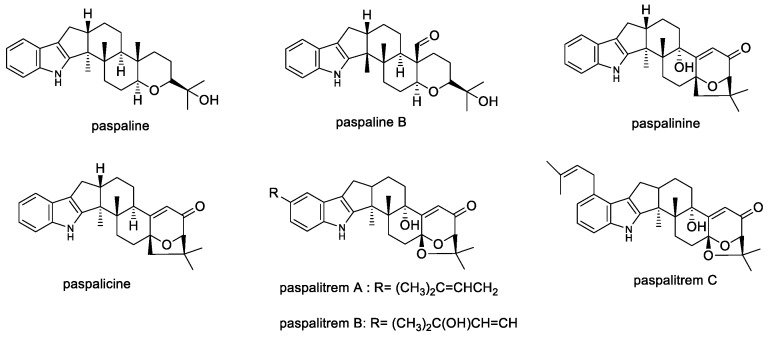
Structures of paspaline and paspaline derivatives.

Paspaline and paspaline B have been isolated from *Pencillium paxilli* [89,90]. Paspaline, paspalinine, and paspalicine (Figure 6) were also isolated from *Claviceps paspali* and all three compounds were shown to possess no toxic or tremorgenic activity [90]. Although, paspalicine [36,89] and paspalitrem C (Figure 6) [65] potently block BK channels (refer to Table 1). This may be related to degradation or structural rearrangement occurring in vivo, thus reducing its potency [41]. 

#### 2.1.5. Terpendoles

**Figure 7:**
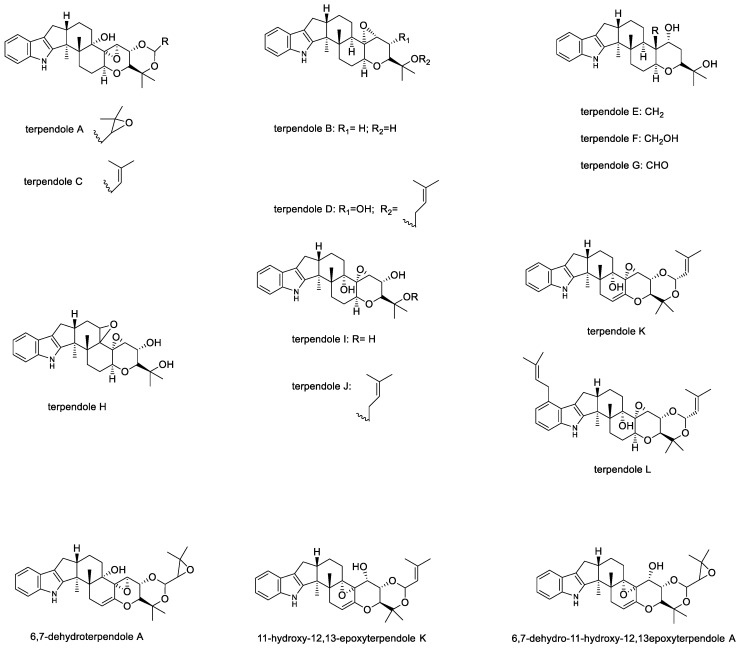
Structures of terpendoles A–L and derivatives.

In 1994, the first reported terpendoles (A–D) (Figure 7) were isolated from culture of the then newly discovered species *Albophoma yamanshiensis* [91]. The compounds paspaline and emindole SB were also reported from this fungus [91]. Terpendoles inhibit acyl-CoA:cholesterol acyl transferase (ACAT) activity in rat liver microsomes [91,92]. The ACAT enzymes are membrane-bound proteins that utilize long-chain fatty acyl-CoA and cholesterol as substrates to form cholesteryl esters. The terpendoles are likely to inhibit ACAT activity by competing with the cholesterol substrate as the two groups exhibit structural similarity. Thus, inhibition of ACAT would be expected to improve or limit the development of atherosclerosis [93]. Terpendole C showed the most potent ACAT inhibition followed by terpendoles D, A, and B [91]. Terpendoles J, K, L and emindole SB exhibited moderate ACAT inhibitory activity, and paspaline and terpendoles E–I showed weak activity [92]. The subsequent identification of two ACAT isoenzymes present in mammals showed that the selectivity of potential inhibitors toward the two ACAT isoenzymes is important for the development of new anti-atherosclerotic agents. Although, terpendole C was found to inhibit both ACAT isoenzymes to a similar extent [94,95]. 

A wide range of microorganisms were selected to test bioactivity of terpendoles A, B, C, and D, and in a separate study, terpendoles E–L [91,92]. The terpendoles did not exhibit any antimicrobial activity [91,92]. However, terpendole E exhibits unique properties; it has been found to be a novel and specific/selective inhibitor of human kinesin, Eg5. In contrast, terpendoles C, H, and I showed no inhibitory activity. Mitotic kinesin activity has been used as a specific target for antitumor compounds [96,97], since cancer therapeutic drugs such as Taxol^®^ (NSC 125973) lack specificity and interfere with other cell functions, causing significant side effects [96]. 

Small animal toxicity studies showed that the terpendole A derivative, 6,7-dehydroterpendole A, elicited the most intense tremors compared to other terpendoles that were tested for tremorgenicity in mice (Table 1) [48]. Terpendole C was found to be a potent and fast-acting tremorgen; terpendole M displayed only weak tremorgenic activity in mice [50]. Since the majority of terpendoles exhibited no toxicological effects on mice, there is an opportunity to further investigate this class of compounds for pharmaceutical and agricultural purposes.

#### 2.1.6. Sulpinines

**Figure 8:**
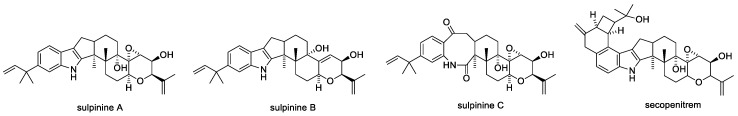
Structures of sulpinines A–C and secopenitrem.

Sulpinines A-C, secopenitrem (Figure 8) and penitrem B (Figure 5) isolated from the sclerotia of *Aspergillus sulphureus* [98] exhibit insecticidal properties and are active against the first instar larvae corn earworm (*Helicoverpa zea*). Feeding trials showed that sulpinine A possesses the strongest bioactivity, observed as reduced weight gain in the insects [98,99]. Sulpinine A also possesses cytotoxicity towards human lung carcinoma, breast adenocarcinoma, and colon adenocarcinoma [98].

#### 2.1.7. Emindoles and Asporyzins

**Figure 9:**
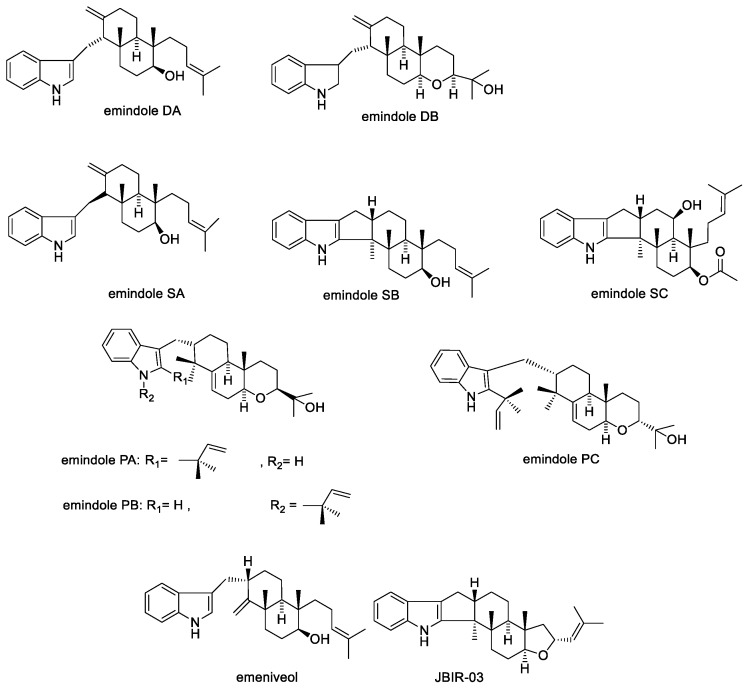
Structures of emindoles.

**Figure 10:**
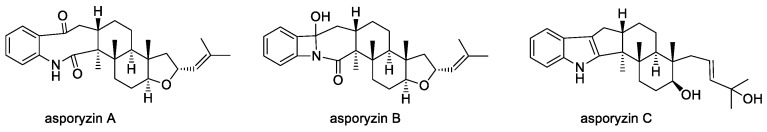
Structures of asporyzins.

Emindoles were first reported in 1987 by Nozawa et al. from the *Emericella* spp. They were named emindole DA, emindole DB, emindole SA, and emindole SB (Figure 9) [68]. Emindoles reported from the mycelium of *Emericella purpurea* include emindoles PA, PB and PC (Figure 9) [100]. Emindole SC (Figure 9) was isolated from *Aspergillus sclerotiicarbonarius* (IBT 28362) and is reported to possess insecticidal properties towards *Drosophila melanogaster* larvae; however, it was found to be inactive against the fungus *Candida albicans* [101]. Emindoles including emindole SB [68] and related compounds emeniveol [102] and JBIR-03 [103] have also been isolated from many other fungal species including *Aspergillus oryzae*, *Dichotomomyces cejpii, Emericella nivea*, and *Emericella striata* [104]. Emeniveol was reported to inhibit pine pollen germination and tea pollen growth [102]. JBIR-03 is reported to have antibacterial activity against methicillin-resistant *Staphylococcus aureus* (MRSA); and also exhibits antifungal activity against the apple Valsa canker-causing fungus, *Valsa ceratosperma* [103]. In a study comparing JBIR-03, emindole SB, emeniveol, and related compounds asporyzin A, asporyzin B, asporyzin C (Figure 10), JBIR-03 was found to possess more potent insecticidal activity against brine shrimp (*Artemia salina*); while asporyzin C showed the most potent antibacterial activity against *Escherichia coli* [104]. 

#### 2.1.8. Aflatrems

**Figure 11:**
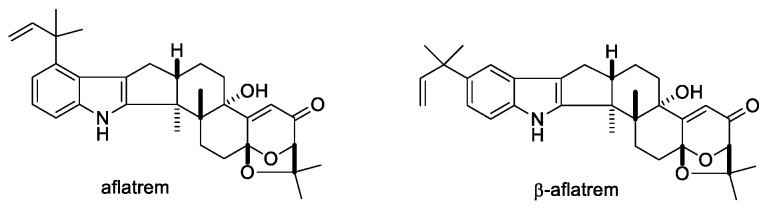
Structures of aflatrems.

Aflatrem and β-aflatrem (Figure 11) are produced by the soil fungus *Aspergillus flavus*. Although, aflatrem is reported as a potent tremorgenic mycotoxin that causes a fast and sharp onset of tremors, it does not exhibit the same level of sustained tremors as lolitrem B (Table 1) [16]. β-aflatrem is reported to cause a significant reduction in the growth rate of the corn earworm *H. zea* [105]. Studies on precursors of aflatrem, such as paspalinine and paspalicine, indicated weaker activity on BK channels compared to aflatrem. Paspalicine an analogue of paspalinine does not exhibit tremorgenic activity but potently blocks BK channels (Table 1) [36]. 

#### 2.1.9. Janthitrems

**Figure 12:**
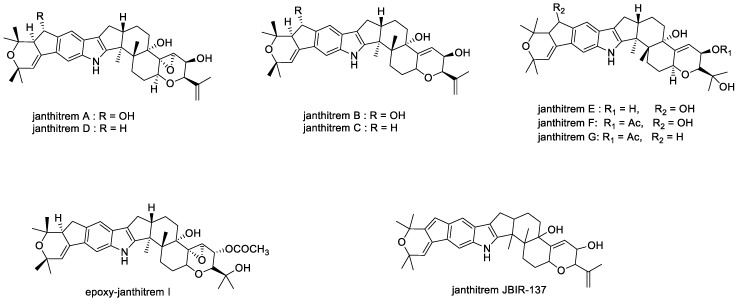
Structures of janthitrems.

In 1980, Gallagher first identified, by high resolution mass spectrometry, the fluorescent tremorgenic mycotoxins janthitrems A, B, and C (Figure 12) in *Penicillium janthinellum* isolates obtained from ryegrass pastures in which ryegrass staggers had been observed [60]. 

Janthitrems B and C were isolated from isolates of *P. janthinellum* as the two most abundant tremorgenic mycotoxins [106]. Full structure elucidation of janthitrem B [106,107] and C [106] were carried out by NMR spectroscopy and later the NMR assignments for janthitrem C were revised [58]. The janthitrems are challenging to isolate as they are reported to be unstable and decompose at 4 °C [59,60]. 

Janthitrem A is reported to be the more potent tremorgen compared to the structurally similar compound janthitrem B (Table 1) [58]. Tremorgenic potency could be attributed to the 11,12-epoxy group as this is the only structural difference [58]. This could explain the tremors observed in animals grazing ryegrass pastures with AR37 endophyte, an *Epichloë* spp. (*Lp*TG-3) strain that is known to produce epoxy-janthitrems [58,108]. Janthitrems A and B also show antifeedant activity against porina (*Wiseana cervinata*) larvae, although janthitrem A is more potent than janthitrem B [58].

Using characteristic properties of the janthitrems, high throughput methods of quantitation were developed in 1982 by Lauren and Gallagher, and a fourth compound was identified as janthitrem D [59,60]. Although in 1984, [109] and later in 1993 [106] janthitrems E–G (Figure 12) was isolated and structures determined, the tremorgenicity of janthitrems E–G remain unknown. 

In 2012, Kawahara et al. described the development of an in-house library by analyzing purified natural compounds from cultures of microorganisms using an ultra-performance liquid chromatography coupled with UV detection (UPLC-UV)-evaporative light-scattering (ELS)-MS system [110]. Compounds which are present in microbial cultures that are registered in the library are automatically identified within the system. The high-throughput screening assay allows the detection of unregistered secondary metabolites in microbial cultures. The *Aspergillus* species was targeted in this scheme due to the large number of bioactive compounds it produced [110]. As a result, a new janthitrem derivative, JBIR-137 was isolated (Figure 12) [110]. The cytotoxic activity reported against human ovarian adenocarcinoma SKOV-3 cell showed that JBIR-137 and janthitrem B exhibit weak cytotoxic activities [110].

#### 2.1.10. Shearinines

**Figure 13:**
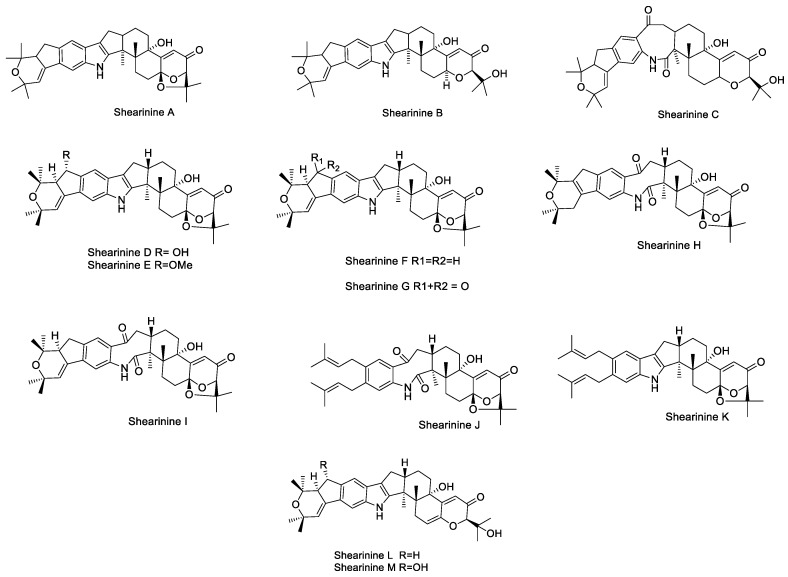
Structures of shearinines A–M**.**

Shearinines are very similar to the janthitrem class of compounds and were first isolated from the aseostromata of *Eupenicillium shearii*. Shearinines A–C (Figure 13) exhibit insecticidal activity as shown in dietary bioassays testing the effect of purified compounds on corn earworm *H. zea* and the dried-fruit beetle *Carpophilus hemipterus* [69]. Shearinine A also exhibited antifeeding activity in a topical assay against *H. zea*, and shearinine B caused significant mortality in the fall armyworm *Spodoptera frugiperda*, when exposed to treated leaf disks [69].

Shearinines D–K have been isolated from *Pencillium* spp., resident endophytic fungus from the mangrove plant *Aegiceras corniculatum* L. Shearinine A, paspalitrem A, and paspaline were also identified. Shearinines D, E, and G (which have reduced potency) showed significant in vitro blocking activity on BK channels [111]. Shearinines also exhibit biological activity against fungal diseases in humans, specifically the *Candida* spp. *Candida albicans* are a common cause of mycoses (fungal diseases) and their ability to form biofilms in human tissues and indwelling medical devices shields the fungus from attack from the immune system and antibiotics [112]. Shearinines D and E have shown to inhibit biofilm formation at a relatively late stage of biofilm development, although they show weaker activity on existing biofilms [112]. Shearinines, in combination with the antifungal drug amphotericin B, augment the potency eight-fold on existing and developing biofilms [112]. 

Shearinines L and M (Figure 13) were isolated from the fungal pathogen *Escovopsis weberi* [113]. Certain ant and fungal species can form mutualistic symbiosis, such as in the case of leaf-cutting ants and their garden fungus (*Leucoagaricus gongylophorus*). The fungal pathogen *E. weberi* was found to degrade the hyphae of *L. gongylophorus* without direct physical interaction by secreting toxins. To investigate the source of the pathogenicity, Dhodary et al. identified and isolated the secondary metabolites produced by the pathogen. The study found that secondary metabolites cycloarthropsone and emodin strongly inhibited the growth of the garden fungus. Shearinine L did not affect the growth of the garden fungus; however, the ants *(Acromyrmex octospinosus*) learned to avoid shearinine L-treated substrate in dual choice behavioural assays [113].

#### 2.1.11. Other Pharmaceutical Applications of Indole-Diterpenes

The indole-diterpenes as active BK channel blockers have opened pharmaceutical applications, including the treatment of glaucoma, a degenerative eye disease that begins with intraocular pressure [114,115,116]. Goetz et al. patented the application of indole-diterpenes for the treatment of glaucoma, as the compounds were shown to reduce intraocular pressure and hence any further degenerative conditions [114,115,116].

A number of indole-diterpenes also show significant activity against the H1N1 virus, particularly emindole SB, 21-isopentenylpaxilline, paspaline, paxilline, and dehydroxypaxilline [117]. The H1N1 virus is an aggressive influenza virus strain that can cause fatalities in humans [117]. To date, there are very few drugs which treat H1N1 viral infections [117].

In summary, there is a common trend to utilize non-tremorgenic indole-diterpenes for pesticides in an agricultural setting. Although, the indole-diterpene group of compounds have proven valuable in applications even beyond the agricultural scope. This could be attributed to their selectivity to receptor binding sites, particularly as BK channel blockers. Minor stereochemical changes have proven to drastically change their effect on the behavioral phenotype in animal models, such as in the case of lolitrem B when it loses its tremorgenicity in its epimeric form (31-epilolitrem B). The unique selectivity exhibited by these compounds have allowed them to be exploited as potential candidates for pharmaceutical drug discovery. However, discrepancies regarding structure–activity relationships remain questionable, particularly in relation to BK channel inhibition and tremorgenicity, which need to be addressed. Lolitrem E and paspalicine, for example, exhibit strong BK channel activity; however, they do not exhibit tremorgenicity. Thus, further investigation would be required into the mode of action of these compounds.

## 3. Toxicity of Ergovaline and Lolitrem B in the Field

The ergot alkaloid largely responsible for mammalian toxicity in tall fescue (*Festuca arundinacea*) pastures is ergovaline—where it accounts for approximately 90% of total plant ergopeptide alkaloid content [10]. Toxicity of ergot alkaloids, although present in endophyte-infected perennial ryegrass, is not as commonly reported as ryegrass staggers disease [118]. The threshold levels for fescue toxicosis is 300–500 parts per billion (ppb), depending on the season [119]. 

There are difficulties in characterizing all compounds in a grass–endophyte association, thus thresholds are set for major toxins. Ergovaline is well studied in tall fescue and has a toxic threshold of 300–400 ppb for cattle and sheep [120]. The Oregon State University College of Veterinary Medicine found the threshold levels of ergovaline in tall fescue, subject to weather, are 300–500 ppb (for horses), 400–750 ppb (for cattle), and 500–800 ppb (for sheep). Threshold levels for lolitrem B in perennial ryegrass staggers for cattle and sheep is 1800–2000 ppb and 500 ppb for camels [119].

In Australia and New Zealand, the concentrations of lolitrem B and ergovaline in naturalized populations of perennial ryegrass are usually lowest in winter/spring from June to November and highest in summer from December to February [19]. A seasonal variation study was carried out by Repussard et al. on lolitrem B and ergovaline concentrations in *Lp*TG-1 infected perennial ryegrass in southern France. Here, concentrations within the leaves, base and inflorescence were analyzed to assess the effect of temperature, rainfall and maturity [120]. Ergovaline was found to accumulate in the inflorescence of the plant whilst lolitrem B varied with the maturity of the plant, whereby concentrations were highest in the oldest leaves. In general, ergovaline and lolitrem B concentrations exhibited similar trends in peaks and troughs (i.e., low or high concentrations), but these similarities vary in different perennial ryegrass varieties/ecotypes [120].

In an effort to identify new host plant–endophyte associations that exhibit nontoxic effects on sheep, Oliveira et al. used two perennial ryegrass genotypes (EI19 and EI24) which were infected with the same lolitrem B-free fungal endophyte [121]. These were sowed on two sites, with different soil types and seasons, in north-west Spain. Low levels of ergovaline were produced and no animal toxicity was reported. A mean value of less than 0.4 ppm ergovaline (mg/kg of dry mass) was reported across the matrix, the highest level being 0.57 ppm [121]. Despite significant seasonal variation in concentrations of ergovaline, grass–endophyte associations safe for agricultural use can be developed. 

## 4. Indole-Diterpenes Reported in the Environment

The presence of these indole-diterpene-type compounds is ubiquitous in the natural environment. Moldy food products produced by the *Penicillium* spp. are a common source for these compounds. For example, fungal metabolites such as janthitrems, paspalinine, paxilline, and 3-*O-*acetoxypaxilline were found to be produced by *Pencillium tularense* found in fresh tomatoes homegrown and from supermarkets, with developing fungal lesions [122]. Also, *Pencillium crustosum* a commonly occurring foodborne fungus that is responsible for spoilage of a wide variety of foods, is known for producing the tremorgenic mycotoxin penitrem A. Humans have developed tremors followed by headache, vomiting, diplopia, weakness, and bloody diarrhea by ingesting *P. crustosum* in moldy food [23] or inhaling moldy hay dust [123]. Also, domestic animals such as dogs exposed to moldy food items have been reported to be poisoned by penitrem A [124,125]. Penitrem A intoxication are characteristic of indole-diterpene intoxication; these include tremors and ataxia that may progress to seizures and death in high levels of exposure. Poisoning has also been documented in cattle, sheep, and horses in the field [24]. 

## 5. High Throughput Methods for Determining Endophyte Toxicity

This review highlights the requirement to screen grass–endophyte associations to determine endophyte toxicity, particularly for novel endophytes that are in the evaluation phase prior to commercialization. Concentrations of metabolites produced by the endophyte, specifically the major toxins lolitrem B and ergovaline, need to be monitored and thoroughly investigated for toxicity to animals. This information is critical for the generation of safer endophyte-infected pastures, and utilizing compounds known to cause limited or no neurotoxic effects whilst still maintaining the competitive advantage for the plant (such as insect control). 

Understanding the toxicity of the indole-diterpene and ergot alkaloid families and their derivatives would provide an avenue in the development of high-throughput screening assays which are required for the selection of novel grass–endophyte associations. Animal models are expensive, time consuming and considered unviable for testing a large number of grass–endophyte associations selected in the breeding program.

In the past, high throughput methods were developed solely based on information on the toxicity of the major mycotoxin lolitrem B. For example, a high-performance liquid chromatography (HPLC) method was developed in 1985 by Gallagher for the quantitation of lolitrem B using the unique profile of 268 nm absorbance of lolitrem B. Although, compounds in the plant matrix with similar UV absorbance’s interfered with the reliable detection of the lolitrems at low levels [126]. Later, Gallagher discovered that the lolitrems had strong fluorescence properties and developed a new sensitive quantitation method using a HPLC fluorescence detector, which remains one of the most sensitive methods used for the detection of lolitrems [127,128]. 

With the advent of metabolomics and mass spectrometry (MS), sample preparation has become much simpler and many compounds can be simultaneously quantitated based on MS and MSMS studies [129]. Currently, liquid chromatography–mass spectrometry (LCMS) is the most widely used tool for identification and quantitation of endophyte-produced alkaloids [10,22,130,131]. However, with the development of facile and rapid methods provided by advanced MS techniques, the limitation now remains on the availability of analytical standards.

In conclusion, current pasture improvement programs involve maintenance of beneficial endophyte infection in perennial ryegrass, whilst reducing the negative effects of particular compounds on mammalian health. It has been long established that lolitrem B and ergovaline, derived from the indole-diterpene and ergot alkaloid pathways, respectively, are the major compounds that cause toxicity syndromes in grazing animals. The contribution of some analogues and intermediates within the indole-diterpene pathway have also more recently been studied for biological activity and mode of action. Currently, information regarding the toxicity status of many of the compounds within the indole-diterpene pathway is lacking, and this information is imperative for the agriculture industry. This is due to naturally varied concentrations of the compounds found in marketed forage grasses. Further, there are limited studies exploring the effect of individual compounds on animal models. The primary challenge is obtaining these compounds through complex isolation and purification methods before any studies can be carried out. Research focused on characterizing the toxicity and biochemical effects of fungal metabolites in animal models will provide greater confidence in dairy and meat products containing these compounds. 

## Figures and Tables

**Table 1:** A summary of the biological activities reported for intermediaries isolated from the indole-diterpene biosynthetic pathway and some synthetic derivatives.

Compound Name	Toxicity as Per Biological Activity on Mice (mg of Compound/kg of Body Weight)	Observation on Mice	Biological Activity on BK/Maxi or *hSlo* Channel	Biological Activity on Animal Model (EMG Activity/Observation in Sheep)	**Reference**
Lolitrem A	2 mg/kg	Severe and prolonged tremor	¯	¯	[44]
Lolitrem B	0.5 to 8.0 mg/kg	Severe and prolonged tremor	IC_50_ = 4 nM (No recovery after wash out)	At 70 µg/kg dose, tremors were observed after 15 min and slowly increased in severity lasting for duration of 12 h. The reticulum and rumen showed inhibition after 20–30 min, coinciding with tremors.	[16,35,41]
31-*epi*-lolitrem B	4 mg/kg	Nontremorgenic	IC_50_ = 50 nM (>50% recovery after 10 min)	The lolitrem B isomer was administered at a dose of 70 µg/kg and there was no effect either on the skeletal muscle EMG activity or EMG activity of the reticulum and rumen.	[33,41,45]
Lolitrem E	2 mg/kg	Nontremorgenic	IC_50_ = 6 nM (No recovery after wash out)	¯	[20,41]
Lolitrem E acetate	16 mg/kg	Weakly tremorgenic	IC_50_ = 2 nM (No recovery after wash out)	¯	[28,41]
31-*epi*-lolitrem F^a^	4 mg/kg	Slightly less tremorgenic than lolitrem B	¯	¯	[46]
Lolitrem F^a^	4 mg/kg	Slightly less tremorgenic than lolitrem B	IC_50_ = 8 nM (No recovery after wash out)	¯	[41,46]
Lolitrem M		Nontremorgenic	IC_50_ = 78 nM (>50% recovery after 10 min)	¯	[41]
Paxilline	4 to 8 mg/kg and an 80 mg/kg dose	Severe but short term tremorgenicity compared to lolitrem B	Complete inhibition = 1 µM (recovery after wash out) Fraction current blocked by 10 nM = 70% (recovery after wash out)	At 1.0 mg/kg dose, moderate to strong tremors; the onset was immediate after 2 min administration; tremors gradually disappeared over the next hour. EMG activity showed both excitatory and inhibitory on the reticulum and rumen. Also, within a minute of infusion, elevations of the EMG activity coincided with induction of marked tremoring.	[33,36,38,47]
13-Desoxypaxilline	8 mg/kg	Nontremorgenic	< 50% inhibition = 30 µM	¯	[38]
α-Paxitriol	100 mg/kg	Lethargy and rough coats, also normal activities such as walking, rearing and preening were greatly reduced for several hours. Animals recovered to normal by 24 h post-injection.	¯	¯	[47]
β-Paxitriol	100 mg/kg	Lethargy and rough coats, also normal activities such as walking, rearing and preening were greatly reduced for several hours. Animals recovered to normal by 24 h post-injection.	¯	¯	[47]
Lolitriol	20 mg/kg	Nontremorgenic	IC_50_ = 196 nM (>50% recovery after 10 min)	¯	[47]
Lolitriol acetate	-	Nontremorgenic	IC_50_ = 43 nM (>50% recovery after 10 min)	¯	[41]
Lolitriol and β-Paxitrol	As a mixture: 16 mg/kg and 100 mg/kg respectively (200 µL dosage)	The single administration of both β-paxitriol and the nontremorgenic lolitriol proved lethal after an initial period of lethargy	-	-	[47]
Lolilline	8 mg/kg	Nontremorgenic	-	-	[43]
6,7-dehydroterpendole A	8 mg/kg	Produced more intense tremors than terpendole C and K at the same dose level	-	-	[48,49]
Terpendole C	4 mg/kg and 8 mg/kg	A fast-acting tremorgen that produced more intense tremors than paxilline, 11-hydroxy12,13-epoxyterpendole K and 6,7-dehydro-11-hydroxy-12, 13 epoxyterpendole A at the same dose level, but the activity ceased after 2 h, as compared to paxilline which ceased after 6 h.	¯	¯	[43,48]
Terpendole D, E, F, G, H and I	8 mg/kg	Nontremorgenic	¯	¯	[28]
Terpendole K	8 mg/kg	Produced more intense tremors than paxilline, 11-hydroxy12,13-epoxyterpendole K and 6,7-dehydro-11-hydroxy-12,13 epoxyterpendole A at the same dose level	¯	¯	[48]
Terpendole M	8 mg/kg	Weakly tremorgenic	¯	¯	[50]
6,7-dehydro-11-hydroxy-12,13 epoxyterpendole A and 11-hydroxy12,13-epoxyterpendole K	8 mg/kg	Mild tremors	¯	¯	[48,49]
Mixture of 88.3% Penitrem A, 6.4% Penitrem B, 5.3% Penitrem E		¯	¯	A dose of 5.5 mg/kg showed no significant skeletal EMG activity, although exhibited strong inhibition on the reticulum and rumen. This was apparent at 15 to 30 min and lasted 2 h. The maximum period of inhibition coincided with the period of greatest tremoring.	[33,51]
Penitrem A	0.75 mg/kg (dose range 0.5 mg/kg to 1.5 mg/kg)	Elicited moderate tremors. Tremor duration reported as several hours.	Fraction current blocked by 10 nM = 100% (no recovery after wash out)	Tremorgenic observation in sheep when given at a dose of 20 µg/kg intravenously.	[36,52,53,54]
Penitrem E	2.25 mg/kg (dose range 1.0 mg/kg to 3.6 mg/kg)	Elicited moderate tremors. Tremor duration reported as several hours. No difference to penitrem A in the rates of onset of tremors observed, and the symptomatology were like-wise similar.	-	-	[54]
Paspaline	¯	¯	Slight inhibition at concentrations up to 1 µM	¯	[38]
Paspalinine	8 mg/kg	Short duration tremors	Fraction current blocked by 10 nM = 100% (no recovery after wash out)	¯	[28,36,55,56]
Paspalicine	250 mg/kg	Nontremorgenic	Fraction current blocked by 10 nM = 83% (recovery after wash out)	¯	[36,55,57]
Paspalitrems	14 mg/kg	Short duration tremors	Fraction current blocked by 10 nM of paspalitrem A and paspalitrem C = 98% and 100% respectively (no recovery after wash out)	¯	[36,55]
Aflatrem	1 mg/kg (dose range 0.5 mg/kg to 4.0 mg/kg)	Short duration tremors	Fraction current blocked by 10 nM = 100% (no recovery after wash out)	¯	[16,36]
Janthitrem A	4 mg/kg	Tremor duration was reported as 8 h and peaked at 15 min. Tremors produced were more intense than janthitrem B, from 2 h post exposure.	¯	¯	[58]
Janthitrem B	6 mg/kg	Tremor duration reported as 6 h and peaked at 30 min. Un-coordination and hypersensitivity to sound and touch is also reported.	¯	¯	[58,59,60]

a) Lolitrem F and 31-*epi*-lolitrem F have been reported to have a similar but slightly reduced tremorgenic activity compared to that reported for lolitrem B; however, Munday-Finch et al. assume that impurities present in lolitrem F and 31-*epi*-lolitrem F could have caused the slightly reduced activity [46].
